# SCA Medium: A New Culture Medium for the Isolation of All *Candida auris* Clades

**DOI:** 10.3390/jof7060433

**Published:** 2021-05-29

**Authors:** Ahmad Ibrahim, Lucie Peyclit, Rim Abdallah, Saber Khelaifia, Amanda Chamieh, Jean-Marc Rolain, Fadi Bittar

**Affiliations:** 1 Aix Marseille University, IRD, APHM, MEPHI, 13005 Marseille, France; ahmad.ibrahim@etu.univ-amu.fr (A.I.); lucie.peyclit@ap-hm.fr (L.P.); rim.abdallah@etu.univ-amu.fr (R.A.); saber.khelaifia@univ-amu.fr (S.K.); amanda.chamieh@etu.univ-amu.fr (A.C.); jean-marc.rolain@univ-amu.fr (J.-M.R.); 2 IHU-Méditerranée Infection, 13005 Marseille, France

**Keywords:** Candida, *Candida auris*, culture, emerging fungus, isolation, specific medium

## Abstract

*Candida auris* is an emerging multidrug-resistant yeast causing nosocomial infections and associated with high mortality in immunocompromised patients. Rapid identification and characterisation are necessary for diagnosis and containing its spread. In this study, we present a selective culture medium for all *C. auris* clades. This medium is sensitive with a limit of detection ranging between 10^1^ and 10^2^ CFU/mL. The 100% specificity of SCA (specific *C. auris*) medium is confirmed on a set of 135 *Candida* strains, 50 bacterial species and 200 human stool samples. Thus, this medium specifically selects for *C. auris* isolation from clinical samples, allowing the latter to study its phenotypic profile.

## 1. Introduction

*Candida auris* is an emerging multidrug-resistant pathogen that was first isolated in 2009 [1] and is now known to have four geographical clades: South Asia: India; East Asia: Japan; Southern Africa: South Africa; and South America: Venezuela [2]. Recently, a potential fifth clade (Clade V) has been described in Iran [3]. *C. auris* is a biofilm-forming, halotolerant, thermo-resistant yeast [1,4] that can grow at temperatures ranging between 30 °C and 42 °C and can tolerate up to 10% salinity [5]. This promotes the ability of *C. auris* to colonize various medical equipment, plastic surfaces, and nosocomial environments [2,3,4], rendering C. *auris* an invasive, powerful pathogen. In addition, *C. auris* is highly resistant to different classes of antifungal agents such as azoles, amphotericin B and echinocandins [6] and is associated with significant mortality, especially in immunocompromised patients with multiple comorbidities such as diabetes mellitus, renal failure, and cardiovascular disease [2,4]. Thus, its rapid identification and characterization are necessary to optimize clinical outcomes and to attempt to contain its nosocomial and worldwide spread.

The diagnosis of *C. auris* is challenging. The vast majority of commercial, readily available diagnostics, such as VITEK, API20C-AUX, Auxa-Color 2, BD Phoenixand MicroScan are misleading. *C. auris* is misidentified as other *Candida* species, namely *Candida famata*, *Candida haemulonii*, *Candida duobushaemulonii*, *Candida sake*, *Candida lustaniae* and *Candida guilliermondii* among others [7,8]. This often leads to delays in appropriate management and treatment. However, the use of Matrix-Assisted Laser Desorption/Ionization Time of Flight Mass Spectrometry (MALDI-TOF-MS) is promising [7,8], since the spectrum of *C. auris* is available in the reference database. In addition, the user can carefully select, “hand-pick”, and isolate the corresponding identified colonies from other colonies growing on a plate. Moreover, the molecular identification and characterization of *C. auris* is well developed [9], and the use of some real-time PCR-targeting *C. auris* and sequencing of the ITS region is promising [9,10,11,12]. In 2017, a modified Sabouraud medium was suggested by Welsh et al. for a specific isolation of *C. auris* [5]. However, some *Candida* isolates were still misidentified [7,8]. Thus, our aim was to develop a specific medium only selective for all *C. auris* clades from clinical samples.

## 2. Materials and Methods

We collected 135 fungal strains (29 different species) [13,14,15] (Table 1) and cultured them on the modified Sabouraud broth suggested by Welsh et al. that contains 20 g/L Mannitol as a carbon source (in order to inhibit *C. glabrata* growth) [5] in both liquid and solid-phase media. For the liquid phase, turbidity of the broth was measured by spectrophotometry at 24, 48, and 72 h of incubation at 40 °C. This served to control the growth of each tested strain. For the solid phase, we added autoclaved bacterial agar (15 g/L) and adjusted for a pH = 7. Each colony was streaked directly on the solid agar or the liquid inoculum.

We then cultured all *C. auris* (*n* = 7) and *Candida tropicalis* (*n* = 6) (see results) strains on the different culture media used in our diagnostic laboratories. The media used for culturing yeasts were: Sabouraud Dextrose Agar, CHROMagar *Candida* and Buffered Charcoal Yeast Extract. The media used for culturing bacteria were: Chocolate agar, Tryptic Soy agar, Columbia agar, Mannitol Salt agar, MacConkey agar (BioMérieux, Marcy-l’Etoile, France) and LBJMR (Lucie Bardet and Jean-Marc Rolain) medium [16].

Finally, we cultured this set of 13 strains (i.e., *C. tropicalis* and *C. auris*) on different combinations of the Welsh et al. broth and MacConkey (MCK) agar, a selective medium for Gram-negative bacteria (see results and discussion) (Table 2) [17]. We prepared several media with varying concentrations of bile salts (1.5 g/L, 1 g/L, 0.75 g/L) and/or crystal violet (0.5 mg/L) (used as inhibitors in MCK) [17] to the initial broth composition of Welsh et al. in solid and liquid phase with shaking (300 rpm) at 40 °C (Table 2).

To study the specificity of our designated medium, we cultured for 3 days at 40 °C a panel of various Gram-positive and Gram-negative bacteria (*n* = 50), different *C. auris* clades (DSM 21092 and 6 strains that were kindly provided by Dr. Jacques F Meis, Canisius Wilhemina Hospital, Department of Medical Microbiology and Infectious Diseases, the Netherlands) [9,18,19,20]. All *C. auris* strains were isolated from blood cultures except JCM 15,448 (Clade II), which is from the external ear canal. We tested also other *Candida* strains (including the closest *Candida* species to *C. auris* (*C. haemulonii* and *C. duobushaemulonii*) (directly streaked on the agar) [2,4]. In addition, we tested 200 fecal samples that were negative by real-time PCR for *C. auris*. These samples were collected at the Marseille Hospital (AP-HM, Assistance Publique-Hôpitaux Marseille) from routine laboratory diagnostics (Table 1). Given that this work did not involve human body/tissues or use clinical data from patients, and according to French law (Loi no 2012–300 of 5 March 2012 and Décret no 2016–1537 of 16 November 2016 published in the ‘Journal Officiel de la République Française’), neither institutional ethical approval nor individual patient consent was required for this non-invasive study.

All patients routinely sign an approval that the samples they submit for testing at AP-HM labs may be used in research. Each stool sample was enriched in Tryptic soy broth (TSB) for 3 days at 37 °C. Then, a total of 0.1 mL of each enriched media was streaked on the solid media. All bacterial and fungal strains used in this work were selected from previous studies and identified correctly by MALDI-TOF-MS [13,14,15,21].

Moreover, to determine the sensitivity of our *C. auris*-specific medium, a series of ten-fold dilution (10^−^^1^ to 10^−^^10^) of 0.5 McFarland of each *C. auris* clade was cultured. For the second clade, we tested the strain of DSMZ collection: DSM 21092. In addition, we also cultured a mixture of the same ten-fold dilutions of 0.5 McFarland of each *C. auris* strain with a stool sample negative in real-time PCR for *C. auris* (10^−^^1^ to 10^−^^10^). We did this as an attempt to determine whether the natural presence of microbes in stools would affect the growth of *C. auris* on our designated medium.

## 3. Results

We first cultured all our available *Candida* spp. strains (*n* = 135) on the broth developed by Welsh et al. Interestingly, we isolated 100% of the tested *C. auris* (*n* = 7) and *C. tropicalis* (*n* = 6) species on the Welsh broth [5].

However, when we cultured all strains on a combination of MacConkey agar and the Welsh broth, the growth of *C. tropicalis* strains was totally inhibited. On the other hand, we observed a normal growth of all *C. auris* species.

**Figure 1:**
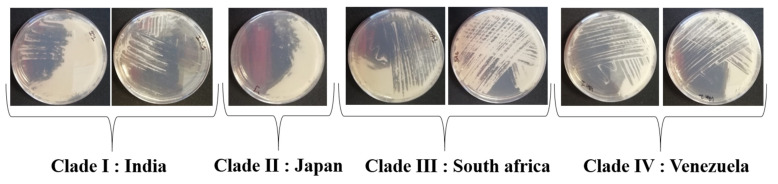
Striking *Candida auris* strains onto SCA medium, 2 strains/clade except for *C. auris* clade II (one strain).

The addition of crystal violet, with or without bile salts, did not affect the growth of *C. auris* (Figure 1), which we evaluated by measuring the turbidity of the broth using a spectrophotometer. However, we observed a total inhibition of all *C. tropicalis* strains in the presence of 0.5 mg/L crystal violet.

The addition of bile salts only partially inhibited the growth of *C. auris* clade II. This was reflected as a decrease in the spectrophotometer value in comparison to the negative control only containing broth and crystal violet. As for *C. tropicalis* strains, the presence of bile salts without crystal violet did not inhibit their growth. It was then excluded from the final composition of the SCA medium (Table 2).

Therefore, the growth of *C. tropicalis* was inhibited by adding crystal violet at 0.5 mg/L to the initial broth suggested by Welsh et al. with no effect on the growth of the 7 *C. auris* strains (2 strains/clade: except for clade II (one strain)) in solid and liquid phase.

Moreover, the specificity of this medium was also confirmed after cultivating all bacterial spp. *Candida* spp. and fecal samples mentioned in Table 1 on the last composition of our medium (Table 3). No growth was noted after 3 days of incubation at 40 °C.

Concerning the sensitivity of our medium, after cultivation of several dilutions of a solution of 0.5 McFarland (10^−1^ to 10^−10^), the limit of detection (LOD) of *C. auris* Clade I, II and VI was 10^2^ CFU/mL in both serial dilutions (with physiological water and a fecal sample). For the third clade (South Africa), we observed an excessive growth of both strains at an LOD of 10^1^ CFU/mL. Thus, the presence of other microbes in a fecal sample does not inhibit or affect the growth of *C. auris*. All resulting colonies were isolated and subsequently identified by MALDI-TOF-MS and real-time PCR [9].

The final composition of our medium in 1 L of deionized water was 5 g of pancreatic digest of casein, 5 g of peptic digest of animal tissue, 20 g of mannitol, 0.5 mg of crystal violet (Sigma-aldrich, Darmstadt, Germany), 100 g NaCl, 50 mg/L of chloramphenicol and 50 mg/L of gentamicin with pH = 7 (±0.2) at 40 °C (Figure 1, Table 3).

## 4. Discussion

Correct identification and rapid isolation of *C. auris* is essential for the timely and appropriate antifungal treatment and for infection prevention and control measures. This ensures limiting and controlling a possible nosocomial outbreak of a pathogen with fatal consequences and limited therapeutic options. This need is emphasized in the current COVID-19 pandemic we are living in, whereby an increasing amount of critically ill, immunocompromised patients populate the hospitals [22,23,24]. The gold standard of diagnosis remains molecular identification by sequencing and/or real-time PCR [9,10,11,12]. However, this is expensive and may only be available at specialized referral or research laboratories. Therefore, we have developed a cheap, simple, easily-prepared medium for isolating *C. auris* that is specific and sensitive.

A caveat to the enrichment broth developed by Welsh et al. for *C. auris* isolation is that it also grows *C. tropicalis* strains, which we demonstrated in our work. We show that the addition of crystal violet (0.5 mg/L) to our specific *C. auris* medium inhibited the growth of *C. tropicalis*. SCA (specific *C. auris*) is considered as a new version of the above-mentioned broth [5].

In addition, other selective media are recently described for the isolation of *C. auris* [25,26,27]. Generally, the selected criteria to cultivate this yeast remain the same as those used in our work: the thermo-resistance, halo-tolerance and multi-resistance to many antifungal agents [2,4]. Here, we managed to develop a medium with the minimum possible inhibitors (40 °C incubation instead of 42, and 10% salinity instead of 12.5% compared to another study [26]). These moderate inhibitors still maintain the selectivity of SCA medium for all *C. auris.*

Usually, *C. auris*, *C. krusei* and *C. parapsilosis* appear as pink colonies on the CHROMagar medium [2,4,28], and a newer version of CHROMagar has been developed to identify *C. auris* [25,27]. This phenotypic identification may be biased and reader-dependent, since it is based on interpretation of the color/morphology of growing colonies. Moreover, a longer duration of incubation may lead to a change in results as well. Thus, a high percentage of error may occur. Our SCA medium facilitates the interpretation of results by only selecting for *C. auris*.

Interestingly, spiking a stool sample with a *C. auris* strain affected neither the specificity nor the sensitivity of this medium. However, increasing our sample size and testing a larger amount of clinical *C. auris* isolates and related yeasts (such as *C. famata, C. pseudohaemulonii, C. metapsilosis, C. orthopsilosis, C. rugosa, C. vulturna, Wickerhamomyces anomalus* and *C. sake*) is necessary for further evaluation, validation and reproducibility of our *C. auris*-specific medium.

## 5. Conclusions

The use of our developed medium enables the rapid, specific isolation of *C. auris* strains and helps in the timely management of patients and resources to limit the occurrence of *C. auris* outbreaks. We propose an implementation of SCA medium in routine clinical mycology for screening skin, urine, vaginal and blood samples, especially in high-risk populations [13,15]. This SCA medium will further enhance our understanding of the phenotypic characteristics of *C. auris* and future isolates, most importantly allowing an accurate study of their antifungal resistance profiles.

## Figures and Tables

**Table 1:** Strains and samples tested on the SCA medium.

Type	Strain/Sample	Nb	Source	Origin	Identification	qPCR *C. auris* (Ibrahim et al., 2021)
Gram-positive bacteria	*Bacillus cereus*	1	Clinical	Marseille, France	MALDI-TOF-MS	Not tested
	*Corynebacterium amycolatum*	1	Clinical	Marseille, France	MALDI-TOF-MS	Not tested
	*Corynebacterium jeikeium*	1	Clinical	Marseille, France	MALDI-TOF-MS	Not tested
	*Corynebacterium propinquum*	1	Clinical	Marseille, France	MALDI-TOF-MS	Not tested
	*Corynebacterium striatum*	1	Clinical	Marseille, France	MALDI-TOF-MS	Not tested
	*Enterococcus faecalis*	1	Clinical	Marseille, France	MALDI-TOF-MS	Not tested
	*Enterococcus faecium*	1	Clinical	Marseille, France	MALDI-TOF-MS	Not tested
	*Micrococcus luteus*	1	Clinical	Marseille, France	MALDI-TOF-MS	Not tested
	*Staphylococcus aureus*	1	Clinical	Marseille, France	MALDI-TOF-MS	Not tested
	*Staphylococcus capitis*	1	Clinical	Marseille, France	MALDI-TOF-MS	Not tested
	*Staphylococcus cohnii*	1	Clinical	Marseille, France	MALDI-TOF-MS	Not tested
	*Staphylococcus epidermidis*	1	Clinical	Marseille, France	MALDI-TOF-MS	Not tested
	*Staphylococcus haemolyticus*	1	Clinical	Marseille, France	MALDI-TOF-MS	Not tested
	*Staphylococcus lugdunensis*	1	Clinical	Marseille, France	MALDI-TOF-MS	Not tested
	*Staphylococcus pasteuri*	1	Clinical	Marseille, France	MALDI-TOF-MS	Not tested
	*Staphylococcus saprophyticus*	1	Clinical	Marseille, France	MALDI-TOF-MS	Not tested
	*Staphylococcus simulans*	1	Clinical	Marseille, France	MALDI-TOF-MS	Not tested
	*Staphylococcus warneri*	1	Clinical	Marseille, France	MALDI-TOF-MS	Not tested
	*Streptococcus agalactiae*	1	Clinical	Marseille, France	MALDI-TOF-MS	Not tested
	*Streptococcus dysgalactiae*	1	Clinical	Marseille, France	MALDI-TOF-MS	Not tested
	*Streptococcus equinus*	1	Clinical	Marseille, France	MALDI-TOF-MS	Not tested
	*Streptococcus mitis*	1	Clinical	Marseille, France	MALDI-TOF-MS	Not tested
	*Streptococcus pneumoniae*	1	Clinical	Marseille, France	MALDI-TOF-MS	Not tested
	*Staphylococcus hominis*	1	Clinical	Marseille, France	MALDI-TOF-MS	Not tested
	*Streptococcus salivarius*	1	Clinical	Marseille, France	MALDI-TOF-MS	Not tested
**Subtotal**		**25**				
Gram-negative bacteria	*Achromobacter xylosoxidans*	1	Clinical	Marseille, France	MALDI-TOF-MS	Not tested
	*Acinetobacter baumannii*	1	Clinical	Marseille, France	MALDI-TOF-MS	Not tested
	*Bacteroides fragilis*	1	Clinical	Marseille, France	MALDI-TOF-MS	Not tested
	*Citrobacter braakii*	1	Clinical	Marseille, France	MALDI-TOF-MS	Not tested
	*Citrobacter freundii*	1	Clinical	Marseille, France	MALDI-TOF-MS	Not tested
	*Citrobacter koseri*	1	Clinical	Marseille, France	MALDI-TOF-MS	Not tested
	*Enterobacter aerogenes*	1	Clinical	Marseille, France	MALDI-TOF-MS	Not tested
	*Enterobacter asburiae*	1	Clinical	Marseille, France	MALDI-TOF-MS	Not tested
	*Enterobacter cloacae*	1	Clinical	Marseille, France	MALDI-TOF-MS	Not tested
	*Enterobacter kobeii*	1	Clinical	Marseille, France	MALDI-TOF-MS	Not tested
	*Escherichia coli*	1	Clinical	Marseille, France	MALDI-TOF-MS	Not tested
	*Haemophilus influenzae*	1	Clinical	Marseille, France	MALDI-TOF-MS	Not tested
	*Haemophilus parainfluenzae*	1	Clinical	Marseille, France	MALDI-TOF-MS	Not tested
	*Hafnia alvei*	1	Clinical	Marseille, France	MALDI-TOF-MS	Not tested
	*Klebsiella oxytoca*	1	Clinical	Marseille, France	MALDI-TOF-MS	Not tested
	*Klebsiella pneumoniae*	1	Clinical	Marseille, France	MALDI-TOF-MS	Not tested
	*Moraxella catarrhalis*	1	Clinical	Marseille, France	MALDI-TOF-MS	Not tested
	*Morganella morganii*	1	Clinical	Marseille, France	MALDI-TOF-MS	Not tested
	*Pasteurella multocida*	1	Clinical	Marseille, France	MALDI-TOF-MS	Not tested
	*Proteus mirabilis*	1	Clinical	Marseille, France	MALDI-TOF-MS	Not tested
	*Proteus vulgaris*	1	Clinical	Marseille, France	MALDI-TOF-MS	Not tested
	*Providencia stuartii*	1	Clinical	Marseille, France	MALDI-TOF-MS	Not tested
	*Pseudomonas aeruginosa*	1	Clinical	Marseille, France	MALDI-TOF-MS	Not tested
	*Raoultella ornithinolytica*	1	Clinical	Marseille, France	MALDI-TOF-MS	Not tested
	*Stenotrophomonas maltophilis*	1	Clinical	Marseille, France	MALDI-TOF-MS	Not tested
**Subtotal**		**25**				
Yeast	*Candida albicans*	73	Clinical	Marseille, France	MALDI-TOF-MS	Not tested
	*Candida glabrata*	8	Clinical	Marseille, France	MALDI-TOF-MS	Not tested
	*Candida krusei*	4	Clinical	Marseille, France	MALDI-TOF-MS	Not tested
	*Candida parapsilosis*	6	Clinical	Marseille, France	MALDI-TOF-MS	Not tested
	*Candida lusitaniae*	3	Clinical	Marseille, France	MALDI-TOF-MS	Not tested
	*Candida tropicalis*	6	Clinical	Marseille, France	MALDI-TOF-MS	Not tested
	*Candida zelanoides*	1	Clinical	Marseille, France	MALDI-TOF-MS	Not tested
	*Candida lipolytica*	1	Clinical	Marseille, France	MALDI-TOF-MS	Not tested
	*Candida inconspicua*	1	Clinical	Marseille, France	MALDI-TOF-MS	Not tested
	*Candida intermedia*	1	Clinical	Marseille, France	MALDI-TOF-MS	Not tested
	*Candida guilliermondii*	3	Clinical	Marseille, France	MALDI-TOF-MS	Not tested
	*Candida bracarensis*	1	Clinical	Marseille, France	MALDI-TOF-MS	Not tested
	*Candida utilis*	1	Clinical	Marseille, France	MALDI-TOF-MS	Not tested
	*Candida bovina*	1	Clinical	Marseille, France	MALDI-TOF-MS	Not tested
	*Candida dubliniensis*	2	Clinical	Marseille, France	MALDI-TOF-MS	Not tested
	*Candida norvegensis*	1	Clinical	Marseille, France	MALDI-TOF-MS	Not tested
	*Candida kefyr*	2	Clinical	Marseille, France	MALDI-TOF-MS	Not tested
	*Candida beverwijkiae*	1	Clinical	Marseille, France	MALDI-TOF-MS	Not tested
	*Cryptococcus diffluens*	1	Clinical	Marseille, France	MALDI-TOF-MS	Not tested
	*Cryptococcus uniguttulatus*	1	Clinical	Marseille, France	MALDI-TOF-MS	Not tested
	*Cryptococcus neoformans*	2	Clinical	Marseille, France	MALDI-TOF-MS	Not tested
	*Saccharomyces cerevisiae*	2	Clinical	Marseille, France	MALDI-TOF-MS	Not tested
	*Rhodotorula mucilaginosa*	1	Clinical	Marseille, France	MALDI-TOF-MS	Not tested
	*Yarrowia lipolitica*	1	Clinical	Marseille, France	MALDI-TOF-MS	Not tested
	*Candida haemulonii*	1	Clinical	Netherlands	MALDI-TOF-MS	Not tested
	*Candida duobushaemulonii*	1	Clinical	Netherlands	MALDI-TOF-MS	Not tested
	*Trichosporon asahii*	1	Clinical	Marseille, France	MALDI-TOF-MS	Not tested
	*Kodamaea ohmeri*	1	Clinical	Marseille, France	MALDI-TOF-MS	Not tested
	*Candida auris* Clade I	2	Clinical	Netherlands	MALDI-TOF-MS	Positive
	*Candida auris* Clade II	1	Clinical	DSMZ collection	MALDI-TOF-MS	Positive
	*Candida auris* Clade III	2	Clinical	Netherlands	MALDI-TOF-MS	Positive
	*Candida auris* Clade IV	2	Clinical	Netherlands	MALDI-TOF-MS	Positive
**Subtotal**		**135**				
Human samples	Stool samples	200	Clinical	Marseille, France		Negative
**Total**		**385**				

**Table 2:** Growth results of *C. auris* (seven strains from four clades) and *C. tropicalis* (*n* = 6) tested strains according to crystal violet and bile salts concentrations for each condition 5 g of pancreatic digest of casein. 5 g of peptic digest of animal tissue, 100 g NaCl, 20 g of Mannitol, 50 mg/L chloramphenicol and 50 mg/L gentamicin were added.

Crystal Violet	Bile Salts	*C. auris* Clade I	*C. auris* Clade II	*C. auris* Clade III	*C. auris* Clade VI	*C. tropicalis*
0 mg/L	0 g/L	++	++	++	++	++
0 mg/L	0.5 g/L	++	++	++	++	++
0 mg/L	1 g/L	++	++	++	++	++
0 mg/L	1.5 g/L	++	+	++	++	+
0.5 mg/L	0 g/L	++	++	++	++	-
0.5 mg/L	0.5 g/L	++	++	++	++	-
0.5 mg/L	1 g/L	++	+	++	++	-
0.5 mg/L	1.5 g/L	++	+	++	++	-

**Table 3:** Final composition of SCA (Specific *Candida auris*) medium.

	Pancreatic Digest of Casein	Peptic Digest of Animal Tissue	NaCl	Mannitol	Crystal Violet	Agar	pH	Chloramphenicol	Gentamicin
Welsh et al. broth	5 g	5 g	100 g	20 g	-	-	5.6	50 mg/L	50 mg/L
SCA medium	5 g	5 g	100 g	20 g	0.5 mg	15 g	7	50 mg/L	50 mg/L

## Data Availability

Not applicable.
